# Increased incidence of teicoplanin-non-susceptible *Staphylococcus epidermidis* strains: a 6-year retrospective study

**DOI:** 10.1038/s41598-023-39666-6

**Published:** 2023-08-03

**Authors:** Subin Kim, Jae-Phil Choi, Dong Hyun Oh, Mi Young Ahn, Eunmi Yang

**Affiliations:** Division of Infectious Disease, Seoul Medical Center, 156, Sinnae-ro, Jungnang-gu, Seoul, 05505 Republic of Korea

**Keywords:** Antimicrobials, Bacteria

## Abstract

Glycopeptide antibiotics (vancomycin and teicoplanin) are usually used for the treatment of *Staphylococcus epidermidis* infections owing to their increased oxacillin resistance. However, *S. epidermidis* strains with decreased susceptibility to teicoplanin have become increasingly incident in recent years. We aimed to identify the characteristics of teicoplanin-non-susceptible (Teico-NS) *S. epidermidis* isolated at our hospital and analyze its relationship with teicoplanin usage. We retrospectively evaluated 328 *S. epidermidis* strains isolated from clinical isolates between January 2016 and December 2021. All strains were susceptible to vancomycin (minimal inhibitory concentration (MIC) ≤ 4 mg/L). The annual incidence for *S. epidermidis* strains with an elevated teicoplanin MIC of 8 mg/L ranged from 22.2 to 28.9%. In addition, in 2021, the number of *S. epidermidis* strains with teicoplanin MIC ≥ 16 mg/L rapidly increased (n = 13, 32.5%). Furthermore, teicoplanin use increased annually until 2019; however, in 2020, it decreased abruptly due to the COVID 19 pandemic. Thus, we could not confirm the existence of a clear correlation between teicoplanin usage and increased incidence of *S. epidermidis* with reduced teicoplanin-susceptibility. We showed the increased incidence of Teico-NS *S. epidermidis* in recent years. Further studies are needed to identify the mechanisms and risk factors for teicoplanin-resistance in *S. epidermidis.*

## Introduction

Coagulase-negative staphylococci (CoNS) constitute a heterogeneous group of bacteria which are important components of normal human skin microbiota^1^. Over the past few decades, they were regarded as nonvirulent contaminants. However, as the number of immunocompromised patients, as well as prosthetic medical device use, has increased, they have become clinically significant as a frequent cause of nosocomial bloodstream infections^1,2^. *Staphylococcus epidermidis* is the most common CoNS species associated with clinically manifested infections^3^.

A large proportion of CoNS nosocomial isolates has been shown to be resistant to multiple antimicrobial agents, including methicillin and other drugs commonly used in treating staphylococcal infections. For this reason, glycopeptide antibiotics (vancomycin and teicoplanin) are often used to treat CoNS infections^1,2^. However, in recent years, CoNS, especially *S. epidermidis*, with elevated minimal inhibitory concentrations (MICs) to teicoplanin (MIC = 8 or ≥ 16 mg/L) have been reported^4–9^. In addition, we detected a rapid increase in the incidence of *S. epidermidis* strains with MIC ≥ 16 mg/L at our hospital in 2021.

Infections caused by *S. epidermidis* with reduced susceptibility to teicoplanin are growing clinical concerns owing to the availability of limited antibiotic options^2,10^. However, data on the recent epidemiological trends of teicoplanin-non-susceptible (Teico-NS) CoNS are limited^4,5,7,8^. Furthermore, the exact mechanisms underlying teicoplanin resistance are still unclear^2^. Therefore, in this study, we aimed to describe the clinical importance of Teico-NS *S. epidermidis* by providing data on recent trends in its incidence over the last 6 years and by analyzing the correlation between this incidence and annual teicoplanin usage.

## Methods

### Study population and design

This study was carried out at Seoul Medical Center, a 650-bed capacity tertiary hospital in Seoul, South Korea. All *S. epidermidis* isolates identified between January 2016 and December 2021 were subjected to microbiological and clinical evaluation. Exclusion criteria were, (1) patients with polymicrobial infections, (2) non-hospitalized patients, (3) strains cultured within 2 days of patient admission, (4) strains cultured repeatedly within 3 months, (5) strains with no susceptibility results, (6) patients aged < 18 years, and (7) patients for which culture test results changed.

We compared baseline characteristics between the teicoplanin-susceptible (Teico-S) and Teico-NS groups and analyzed the glycopeptide MICs of all strains with respect to the year. In addition, we analyzed the relationship between the annual teicoplanin usage and the incidence of *S. epidermidis* with elevated teicoplanin MIC.

### Data collection

Demographic, clinical, and microbiological data were reviewed retrospectively from medical records. Data were collected on patient age, sex, underlying diseases, previous antimicrobial treatment using vancomycin or teicoplanin, source of isolates, and antimicrobial susceptibility test results to oxacillin, teicoplanin, and vancomycin. In addition, annual teicoplanin and vancomycin usage in the hospital was investigated.

### Definitions

The Teico-S and Teico-NS groups were defined as *S. epidermidis* strains with MIC < 16 mg/L and ≥ 16 mg/L, respectively. Oxacillin resistance was defined as oxacillin MIC ≥ 0.5 mg/L in *S. epidermidis* strains. Annual teicoplanin and vancomycin usage was expressed as annual defined daily doses (DDDs) per 1000 occupied bed days (OBD), according to the Anatomical Therapeutic Chemical Classification/DDD System defined by the World Health Organization^11^.

### Antimicrobial susceptibility testing

All isolates were identified using the Microscan system (MicroScan WalkAway-96 Plus, Siemens, Deerfield, IL, USA) and a matrix-assisted laser desorption ionization-time of flight mass spectrometry system (Bruker Daltonik GmbH, Bremen, Germany). Antimicrobial susceptibility tests were performed using the Microscan and VITEK® 2 systems (bioMérieux, Marcy l'Etoile, France), and MIC values were reported in mg/L.

The susceptibility categories of the MIC values obtained were interpreted according to the Clinical and Laboratory Standards Institute (CLSI) guidelines^12^. Teicoplanin MIC values for *S. epidermidis* were interpreted as follows: < 16 mg/L was considered susceptible and ≥ 16 mg/L was considered non-susceptible (specifically, 16 mg/L was considered intermediate and ≥ 32 mg/L was considered resistant). Vancomycin MIC values for *S. epidermidis* were interpreted as follows: < 8 mg/L was considered susceptible and ≥ 8 mg/L was considered resistant.

### Statistical analysis

Age and MIC were expressed using the median and interquartile range (IQR). Student’s *t*-test was used to compare patient age between the Teico-S and Teico-NS groups. Discrete variables were expressed as frequencies or percentages. Between-group comparisons were conducted via univariate analysis using the χ^2^ test and Fisher's exact test. *P* values < 0.05 were considered statistically significant. Statistical analyses were performed using SPSS version 26.0 (Statistical Product Inc., Chicago, IL, USA).

### Ethics declarations

The study protocol was approved by the Institutional Review Board (IRB) of Seoul Medical Center in 2022 (SEOUL 2022-03-002-002). In addition, informed consent for this retrospective study, which was based on patient electronic medical records, was waived by the IRB of Seoul Medical Center. Furthermore, the study was performed within the confines of the tenets of the Declaration of Helsinki.

## Results

**Figure 1:**
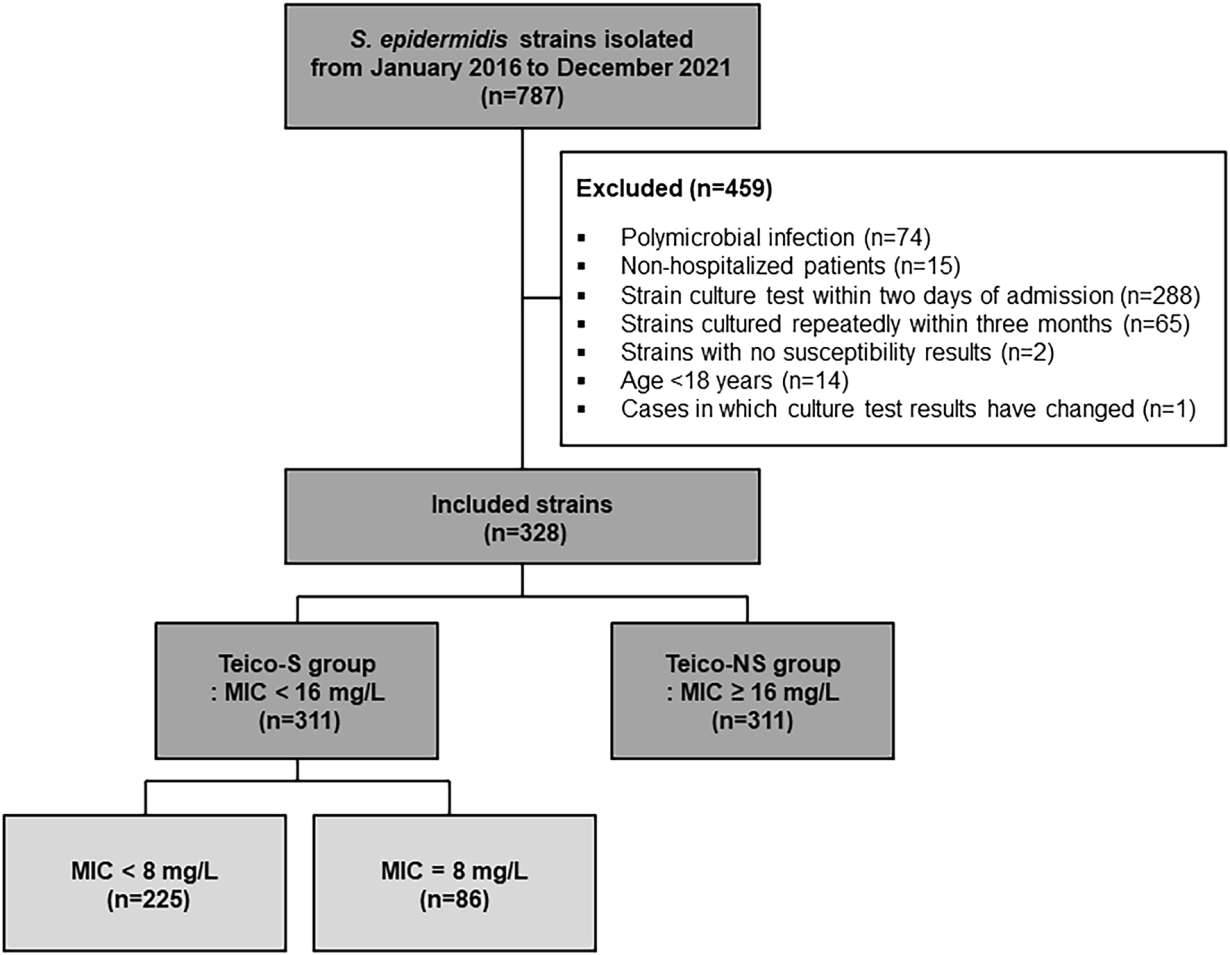
Flow chart of the study design. *Teico-S* teicoplanin-susceptible, *Teico-NS* teicoplanin-non-susceptible, *MIC* minimal inhibitory concentration.

Within the study period, a total of 787 *S. epidermidis* strains were isolated at our hospital. Of these, 328 strains, from 323 patients, were included in the study. Of the 328 strains included in the study, 17 (5.2%) and 311 (94.8%) were classified into the Teico-S and Teico-NS groups, respectively (Fig. 1). Blood samples were the most common infectious specimens collected in this study, accounting for 87.2% of the cases, followed by wound specimens, ascites fluid, pleural fluid, Jackson–Pratt drains, abscesses, cerebrospinal fluid, and central venous catheter tips.

A comparison of the clinical characteristics of strains in the Teico-NS and Teico-S groups is shown in Table 1. Patients in the Teico-NS group were significantly older than those in the Teico-S group (77 [70.5–83.0] years vs. 68 [57.0–79.0] years; *P* = 0.01). The proportion of patients with diabetes mellitus was higher in the Teico-NS group than in the Teico-S group (52.9% and 37.6%, respectively, *P* = 0.21). A total of 14 patients infected with COVID-19 were included in this study. In addition, the prevalence of COVID-19 infection was significantly higher in the Teico-NS group than in the Teico-S group (29.4% vs. 2.9%, *P* = 0.04). A total of 40 (12.2%) and 105 (32%) patients were treated with vancomycin and teicoplanin, respectively. There was no statistically significant difference in the rate of previous glycopeptide use between the two groups (*P* = 0.31). In the Teico-NS group, no patient was previously treated with vancomycin, whereas four (23.5%) were previously treated with teicoplanin. All strains in the Teico-NS group showed oxacillin resistance. The prevalence of oxacillin resistance was higher in the Teico-NS group than in the Teico-S group; however, this was not statistically significant (*P* = 0.23).

**Table 1:** Comparison of baseline characteristics of patients infected with *S. epidermidis* isolates according to their teicoplanin-susceptibility.

Characteristic, N (%)	Teico-S group^a^ (n = 311)	Teico-NS group^b^ (n = 17)	*P* value
Demographics			
Age, median (IQR), years	68.0 (57.0–79.0)	77.0 (70.5–83.0)	0.01
Male	187 (60.1)	9 (52.9)	0.62
Underlying disease			
Hypertension	165 (53.1)	8 (47.1)	0.63
Diabetes mellitus	117 (37.6)	9 (52.9)	0.21
Congestive heart disease	16 (5.1)	0	1.00
Cerebrovascular accident	85 (27.3)	2 (11.8)	0.26
Chronic liver disease	21 (6.8)	1 (5.9)	1.00
Chronic kidney disease	29 (9.3)	1 (5.9)	1.00
Solid cancer	55 (17.7)	1 (5.9)	0.32
Hematological malignancy	4 (1.3)	1 (5.9)	0.24
COVID-19 infection^c^	9 (2.9)	5 (29.4)	0.04
Previous antibiotic use	273 (87.8)	15 (88.2)	1.00
Glycopeptide antibiotics	122 (39.2)	4 (23.5)	0.31
Vancomycin	40 (12.9)	0 (0.0)	0.24
Teicoplanin	101 (32.5)	4 (23.5)	0.60
Source of isolates			
Blood	236 (81.1)	15 (88.2)	0.36
Oxacillin-resistance	275 (88.4)	17 (100.0)	0.23

Data are presented as the number of patients (with the corresponding percentage in parentheses) unless otherwise specified.

*Teico-S* teicoplanin-susceptible, *Teico-NS* teicoplanin-non-susceptible, *IQR* interquartile range, *MIC* minimal inhibitory concentration.

^a^Teicoplanin MIC values < 16 mg/L.

^b^Teicoplanin MIC values ≥ 16 mg/L.

^c^Patients infected with COVID-19 were only present in 2020 and 2021. (n = 6 and 8, respectively).

Table 2 shows data on vancomycin and teicoplanin MIC values for *S. epidermidis* strains with respect to the year. All 328 strains were susceptible to vancomycin. The proportion of isolates with a teicoplanin MIC value of 8 mg/L remained constant, at approximately 20%. One (1.2%) strain isolated in 2016 was non-susceptible to teicoplanin (MIC ≥ 16 µg/mL), whereas three strains (5.0%) isolated in 2017 were non-susceptible to teicoplanin. From 2017 to 2020, Teico-NS strains were not isolated at the hospital. However, in 2021, the number of Teico-NS strains isolated at the hospital significantly increased to 13 (32.5%). In addition, the median teicoplanin MIC value between 2016 and 2020 was 4 mg/L, but increased to 8 mg/L in 2021.

**Table 2:** Antimicrobial susceptibility test results for teicoplanin and vancomycin in *S. epidermidis* from 2016 to 2021.

	Year					
	2016	2017	2018	2019	2020	2021
Glycopeptide antibiotic MICs, median (IQR) (mg/L)						
Teicoplanin	4 (4–8)	4 (4–8)	4	4 (2–8)	4 (2–8)	8 (4–16)
Vancomycin	2 (1–2)	2 (1–2)	2	2 (1–2)	2 (1–2)	1 (1–2)
Number of isolates with respect to teicoplanin MIC values, N (%)						
MIC < 16 mg/L^a^						
MIC < 8 mg/L	58 (69.9)	42 (77.8)	56 (77.8)	40 (72.7)	13 (72.2)	16 (40.0)
MIC = 8 mg/L	24 (28.9)	15 (25.0)	16 (22.2)	15 (27.3)	5 (27.8)	11 (26.5)
MIC ≥ 16 mg/L^b^	1 (1.2)	3 (5.0)	0	0	0	13 (32.5)

*Teico-S* teicoplanin-susceptible, *Teico-NS* teicoplanin-non-susceptible, *MIC* minimal inhibitory concentration, *IQR* interquartile range.

^a^Teico-S group.

^b^Teico-NS group.

**Figure 2:**
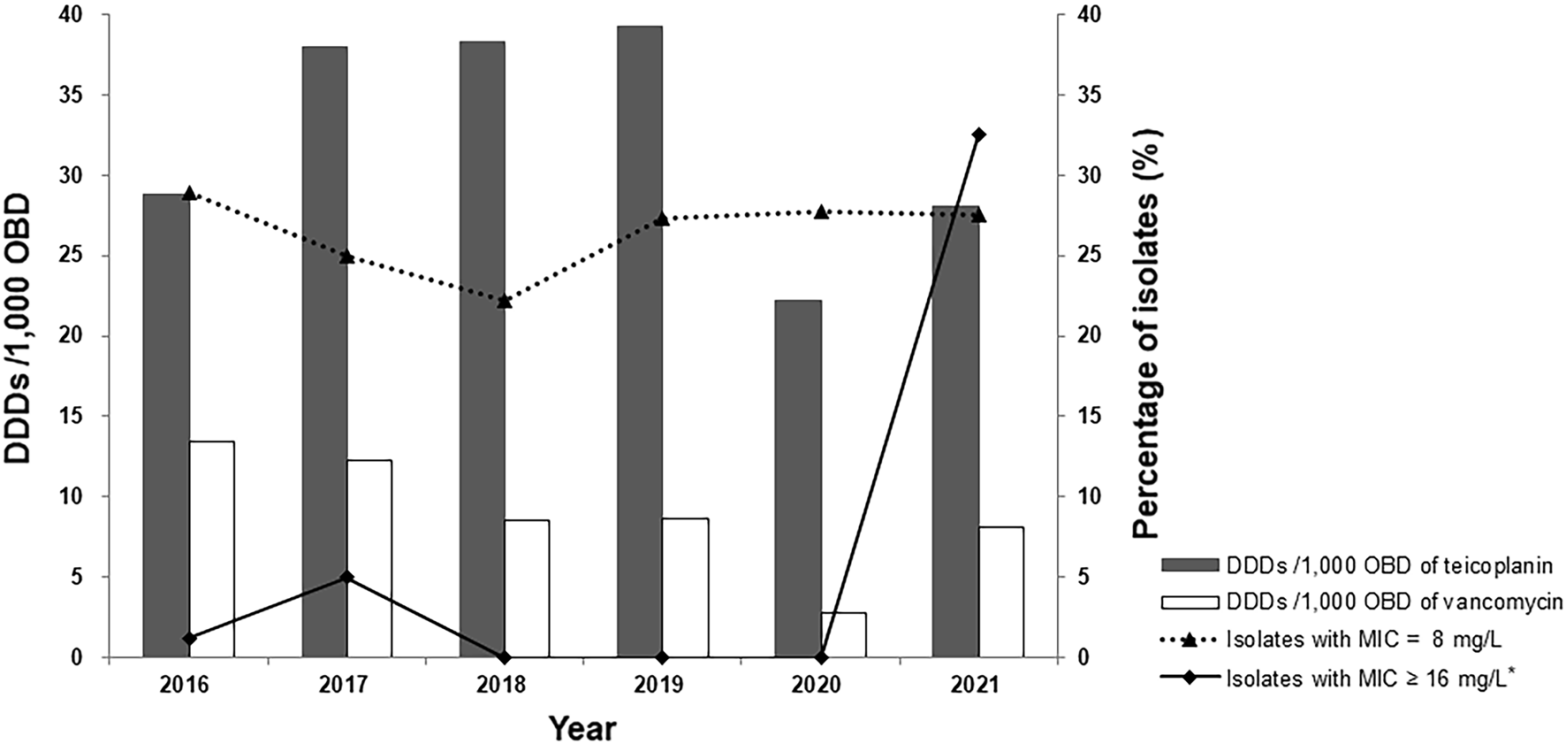
Relationship between the incidence *of S. epidermidis* with elevated MIC values (8 or ≥ 16 mg/L) and annual glycopeptide antibiotic usage in DDDs/1000 OBD. *Teico-NS group. *DDD* defined daily dose, *OBD* occupied bed days, *MIC* minimal inhibitory concentration.

We also analyzed annual glycopeptide antibiotic usage at our hospital (Fig. 2). The annual teicoplanin prescription rate was higher than that of vancomycin throughout the study period. Annual teicoplanin usage continuously increased from 2016 to 2019 i.e., 28.8 DDDs/1000 OBD in 2016 and 39.3 DDDs/1000 OBD in 2019. However, it decreased rapidly to 22.2 DDDs/1000 OBD in 2020. In this study, the correlation between annual teicoplanin usage and the incidence of *S. epidermidis* with elevated teicoplanin MIC values (≥ 16 mg/L) was not confirmed.

## Discussion

*Staphylococcus epidermidis*, the most frequently isolated CoNS species, is an important cause of various healthcare-associated infections, such as venous catheter-related bloodstream infection and prosthetic valve endocarditis, owing to its biofilm-forming properties^2,13^. In this study, we found the median teicoplanin MIC value for *S. epidermidis* strains to increase from 4 to 8 mg/L in 2021, and the incidence of Teico-NS (MIC ≥ 16 mg/L) strains increased dramatically to 32.5% (n = 13) in the same year. We could not demonstrate the existence of a clear correlation between annual teicoplanin usage and the increased incidence of Teico-NS strains.

Several studies carried out in different parts of the world have also reported the isolation of CoNS with decreased teicoplanin susceptibility^5,7,14–16^. Wijesooriya et al.^7^ isolated CoNS strains with decreased teicoplanin susceptibility (MIC ≥ 16 mg/L) from 1510 isolates (7.2%) between 2010 and 2012 in the Australian healthcare network. In addition, Kresken et al.^5^ detected CoNS strains with teicoplanin MIC values ≥ 8 mg/L in 10.6% of a total of 630 isolates. However, most CoNS isolates identified in these previous studies were found to still be susceptible to vancomycin^2,5,7,13^. In this study, we also found all identified CoNS strains to be susceptible to vancomycin, with MIC values less than 8 mg/L.

The exact mechanism underlying teicoplanin-resistance in CoNS remains unclear^2^. Biavasco et al.^17^ reported that glycopeptide resistance in staphylococcal strains may have an endogenous mechanism as glycopeptide-resistant cells have shown several different features from glycopeptide-susceptible cells, including ultrastructural morphology, glycopeptide-binding capacity, number of membrane proteins, cell wall composition, and susceptibility to cell wall-active antibiotics and enzymes. For instance, in a study carried out on teicoplanin-resistant and vancomycin-susceptible CoNS clinical isolates, O’Hare and Reynolds^18^ demonstrated the presence of a 39-kDa protein in the membrane of a resistant *S. epidermidis* strain; this protein was either absent in susceptible control strains of the same species or present at significantly low levels.

Some studies have found that previous glycopeptide use in individual patients may be correlated with teicoplanin resistance^7,19^. However, in our study, there was no significant difference between the Teico-S and Teico-NS groups with respect to previous glycopeptide use (39.2% vs. 23.5%; *P* = 0.31) (Table 1). Clonal spread is a known potential cause of the transmission of multidrug-resistant CoNS in hospital settings^2^. We suspect that in this study, nosocomial transmission may have affected the isolation of Teico-NS strains in patients without a previous history of glycopeptide use.

Furthermore, a previous study demonstrated a significant correlation between the incidence of Teico-NS (MIC ≥ 8 mg/L) CoNS strains and vancomycin use (correlation coefficient: 0.77, *P* ≤ 0.001), as well as a moderate correlation between this incidence and teicoplanin use (correlation coefficient: 0.42, *P* = 0.001)^16^. They suggested that the role of teicoplanin in the selection of resistant strains may have been masked because their hospital used less teicoplanin than vancomycin. Considering this, we speculated that antibiotic selection pressure through extensive glycopeptide antibiotic use might contribute to decreased teicoplanin susceptibility in *S. epidermidis*.

Thus, we sought to determine whether there was an association between teicoplanin use and the incidence of Teico-NS *S. epidermidis* at our hospital. Although annual teicoplanin use increased from 2016 to 2019 at our hospital, it decreased abruptly in 2020, and we could not identify a clear correlation between this usage and the incidence of CoNS strains with reduced teicoplanin susceptibility. This is because owing to the COVID-19 pandemic in Korea, our hospital was designated as a national isolation center for COVID-19 patients from January 2020 to May 2022. Approximately 7000 patients were hospitalized for COVID-19 infection from 2020 to 2021. In 2020, the number of hospitalizations for non-COVID-19 diseases decreased by 60% from the average annual number of hospitalizations of about 180,000 from 2016 to 2019. As a result, OBD values in 2020 decreased to approximately half of the average OBD values obtained from 2016 to 2019 (81,291 and 184,303 days, respectively).

Furthermore, we found that the percentage of isolates with an MIC value of 8 mg/L remained constant at approximately 20% during the study period. In this study, these isolates were classified as Teico-S strains; however, based on the European Committee on Antimicrobial Susceptibility Testing (EUCAST) guidelines, they could be defined as teicoplanin resistant strains^20^. Therefore, as the teicoplanin-resistance threshold varies with respect to the criteria, we considered that analysis of isolates with an MIC of 8 mg/L would be clinically meaningful. Considering that some studies have reported the presence of CoNS with teicoplanin heteroresistance^15,17^, in this study, we speculated that isolates exhibiting an MIC of 8 mg/L might have this property. Based on this speculation, the distribution of MIC values for isolates might have changed as teicoplanin-resistant subpopulations became dominant due to antibiotic selection pressure. However, to validate our hypothesis, further studies to confirm heteroresistance via population analysis profiling are needed^17^.

In addition, the Teico-NS group had a greater proportion of older and COVID-19 infected patients than the Teico-S group. We speculate that age and COVID-19 infection could be risk factors for reduced teicoplanin susceptibility in CoNS, and multiple mechanisms may underly glycopeptide resistance in CoNS strains. Therefore, further large-scale clinical studies involving other CoNS strains, as well as *S. epidermidis* strains, need to be carried out to identify risk factors for teicoplanin resistance in CoNS.

Antibiotic options for the treatment of infections caused by *S. epidermidis* with decreased teicoplanin susceptibility are highly limited. As mentioned above, as susceptibility to vancomycin has been shown in several studies to remain relatively constant^2,5,7,13^, clinicians may consider vancomycin as a preferred alternative antibiotic. However, in patients with acute kidney injury, the use of vancomycin is limited due to its renal toxicity. In such patients, daptomycin and linezolid could be used as alternatives. Although susceptibility to these antibiotics has been shown to be conserved^6^, some studies have reported their resistance in some CoNS strains^21^. Therefore, further research on newer antibiotics is urgently needed, along with more active surveillance of the use of existing antibiotics.

Our study had some limitations. First, our findings are based on patient data collected retrospectively from a single healthcare institution. Second, we analyzed the clinical characteristics and resistance status of *S. epidermidis* strains only. To determine the specific cause of teicoplanin resistance in CoNS, further studies that will involve not only *S. epidermidis* strains, but other CoNS strains also need to be carried out. Third, to include only nosocomial *S. epidermidis* isolates in this study, we excluded specimens collected from emergency rooms and outpatient clinics, as well as those collected within 2 days of hospitalization. Therefore, our results may not reflect CoNS resistance in community settings. Fourth, we did not reveal the specific dose and duration of antibiotics used previously and the reason for antibiotic use, and these factors may have influenced the increase in resistance.

In conclusion, we demonstrated the increased incidence of *S. epidermidis* strains with elevated teicoplanin MIC values over a 6-year period at our hospital. Given the impact of the COVID-19 pandemic, it is necessary to conduct additional time series analyses, which should include data from 2021 onwards, to evaluate the correlation between teicoplanin use and increased Teico-NS CoNS incidence. In addition, an established antibiotic stewardship program should be implemented, and strict monitoring of CoNS resistance status carried out.

## Data Availability

Data that support the findings of this study are available upon reasonable request to the corresponding author.

## References

[1] Rupp ME, Fey PD, John EB, Raphael D, Martin JB (2020). Mandell, Douglas, and Bennett’s Principles and Practice of Infectious Diseases.

[2] Becker K, Heilmann C, Peters G (2014). Coagulase-negative staphylococci. Clin. Microbiol. Rev..

[3] European Centre for Disease Prevention and Control (2018). Multidrug-Resistant Staphylococcus epidermidis.

[4] Baris A (2020). Evaluation of teicoplanin resistance detected by automated system in coagulase negative staphylococci: A comparison with gradient test and broth microdilution methods. Curr. Microbiol..

[5] Kresken M (2022). Glycopeptide resistance in *Enterococcus* spp. and coagulase-negative staphylococci from hospitalised patients in Germany: Occurrence, characteristics and dalbavancin susceptibility. J. Glob. Antimicrob. Resist..

[6] Natoli S (2009). Characterization of coagulase-negative staphylococcal isolates from blood with reduced susceptibility to glycopeptides and therapeutic options. BMC Infect. Dis..

[7] Wijesooriya W, Kotsanas D, Korman T, Graham M (2017). Teicoplanin non-susceptible coagulase-negative staphylococci in a large Australian healthcare network: Implications for treatment with vancomycin. Sri Lankan J. Infect. Dis..

[8] Mehri H (2017). Investigation of glycopeptide susceptibility of coagulase-negative staphylococci (CoNS) from a tertiary care hospital in Gorgan, northern Iran. Arch. Pediatr. Infect. Dis..

[9] Trueba F (2006). High prevalence of teicoplanin resistance among *Staphylococcus*
*epidermidis* strains in a 5-year retrospective study. J. Clin. Microbiol..

[10] Centers for Disease Control and Prevention. *Healthcare-Associated Infections (HAIs)-Laboratory Detection of Coagulase-Negative Staphylococcus species with Decreased Susceptibility to the Glycopeptides Vancomycin and Teicoplanin.* https://www.cdc.gov/hai/settings/lab/labdetectioncoagulase_negative.html (2019). Accessed 16 June 2023.

[11] World Health Organization. *Definition and General Considerations: Defined Daily Dose (DDD).* https://www.whocc.no/ddd/definition_and_general_considera/ (2018). Accessed 16 June 2023.

[12] CLSI. *Performance Standards for Antimicrobial Susceptibility Testing, 30th Edition. CSLI Supplement M100* 1-294 (2020).

[13] Franca A, Gaio V, Lopes N, Melo LDR (2021). Virulence factors in coagulase-negative staphylococci. Pathogens.

[14] Ma XX, Wang EH, Liu Y, Luo EJ (2011). Antibiotic susceptibility of coagulase-negative staphylococci (CoNS): Emergence of teicoplanin-non-susceptible CoNS strains with inducible resistance to vancomycin. J. Med. Microbiol..

[15] Nunes AP (2007). Heterogeneous resistance to vancomycin and teicoplanin among *Staphylococcus* spp. isolated from bacteremia. Braz. J. Infect. Dis..

[16] Bertin M (2004). Relationship between glycopeptide use and decreased susceptibility to teicoplanin in isolates of coagulase-negative staphylococci. Eur. J. Clin. Microbiol. Infect. Dis..

[17] Biavasco F, Vignaroli C, Varaldo PE (2000). Glycopeptide resistance in coagulase-negative staphylococci. Eur. J. Clin. Microbiol. Infect. Dis..

[18] O'Hare MD, Reynolds PE (1992). Novel membrane proteins present in teicoplanin-resistant, vancomycin-sensitive, coagulase-negative *Staphylococcus* spp. J. Antimicrob. Chemother..

[19] Maugein J (1990). In vitro activities of vancomycin and teicoplanin against coagulase-negative staphylococci isolated from neutropenic patients. Antimicrob. Agents Chemother..

[20] EUCAST. *Clinical Breakpoints-Breakpoints and Guidance. Version 13.0*. https://www.eucast.org/clinical_breakpoints (2023). Accessed 15 June 2023.

[21] Watanabe S (2019). Association between daptomycin susceptibility and teicoplanin resistance in *Staphylococcus*
*epidermidis*. Sci. Rep..

